# Resilience analysis of the local communities from a political economy perspective in Zanjan, Iran

**DOI:** 10.1038/s41598-023-46838-x

**Published:** 2023-11-08

**Authors:** Saeid Zarghami, Lotfali Kozehgar Kaleji, Maryam Abhari

**Affiliations:** 1 Faculty of Earth Sciences, Shahid Beheshti University, Tehran, Iran; 2 Faculty of Earth Sciences, Human Geography and Spatial Planning Department, Shahid Beheshti University, Tehran, Iran

**Keywords:** Environmental economics, Psychology and behaviour, Sustainability

## Abstract

Local community resilience has been a solution to reduce human and natural origin damages for several decades in global studies. Various studies have addressed different aspects of resilience. However, using the results of this research to create local community resilience has always faced limitations. In fact, the neglected role of political economy in these studies has caused the application of policies and strategies resulting from these studies to face challenge to create local communities’ resilience. Therefore, the aim of this research is to evaluate how political economy affects the resilience of local communities. The research method is mixed and qualitative analysis was used to analyze the impact of political economy on resilience and quantitative analysis was used to measure the extent of resilience. The research has investigated the impact of political economy on the aspects of resilience and measured the extent of resilience from economic, social, institutional, environmental, and physical aspects by selecting the central part of Zanjan. The results of the research show that political economy has caused the lack of role-playing of local communities in resilience creation. In fact, policy-making influenced by political economy has led to the formation of a rent economy, a top-down and centralized management system, and this was the main obstacle in creating resilient local communities directly and indirectly through the reduction of the role of local institutions, spatial segregation, housing rent, reduction of social capital, increasing greenhouse gases and poverty. Therefore, we need a transition from the current institutional environment take steps towards evolution, dynamism, and institutional transformation to create local communities’ resilience in order to create a resilient local society.

## Introduction

Cities are complex and interdependent systems that are vulnerable to threats caused by natural and human disasters. Although communities can predict some of the consequences of hazards, many of the effects are unknown and unpredictable^1,2^. Therefore, in the past few decades, resilience has been considered as a new paradigm among development organizations (including non-governmental and non-profit organizations) to survive in a turbulent and changing world^3^. Resilience is the ability of a system, society, or community faced with risks to sustain, absorb, and adapt to it and recover from the effects of risk in an efficient and timely manner, including through preserving and restoring its fundamental structures and functions^4–9^. Therefore, resilience is a useful concept beyond social and natural concepts and has been increasingly used in policymaking^10^. Researchers in the field of environmental issues have used resilience to study human societies. In recent years, a new perspective on resilience has emerged. Concepts such as community capacities and potentials, creating knowledge and awareness networks, which are referred to as influential forces in social resilience, have been introduced^11–15^. Therefore, various research has been conducted on resilience from different aspects, and most of the research has focused on the resilient community and its criteria. Additionally, various organizations have provided frameworks for measuring resilience, using quantitative and qualitative methods in most of the studies^16^.

A review of the literature indicates two general categories in relation to the resilience discussion. The first category is the evaluation of ecological resilience, which mainly focuses on the analysis of systems and ecosystems^1,17–19^. The second category is related to studies on social resilience. Resilience from individuals to society, especially in the psychological dimension, is more about the social environment and issues such as the threshold of individuals’ tolerance. Another aspect is the ecologically-social approach, which, in addition to emphasizing environmental issues, also emphasizes the social environment and considers resilience in the continuity of the human community with ecosystem services and resources for social sustainability. This approach aims to apply the models and methods used for ecological resilience to social issues as well^20–24,24–26^. However, this approach also faces serious challenges. The first challenge is that ecological resilience does not necessarily lead to social resilience. On the other hand, some researchers believe that the mechanism of environmental system elements is not generalizable to the social system. Although there are similarities, the feedback process of the social system is also affected by actors in addition to the structure^27^. In addition to the overall approach of ecological resilience, all studies have focused on the system’s ability to return after disruption, while in social resilience, crises are identified as an opportunity for creating change and development^28^. Some researchers consider the requirements of resilience to be linked to social, economic, and environmental capital, and believe that addressing these three paths of capital leads to an increase in resilience in society^10,29^.

In general, the concept of resilience has evolved over time and new concepts have also been developed related to it. The study of world literature indicates that the paradigm has changed from equilibrium models into non-equilibrium ones. Resilience models are divided into equilibrium (quick return of system to normal state) and non-equilibrium ones^30^. The non-evolutionary model (evolutionary resilience) is based on the assumption that social-ecological systems respond differently depending on the type of disturbance, stress, and spatial diversity. Unlike the equilibrium model, which emphasizes the entire system to return to normal status, the non-equilibrium one pays attention to one of the system’s capacities and adapting to conditions instead of emphasizing the vulnerability reduction^31^. Recently, most researches in the field of resilience are based on non-equilibrium models that rely on concepts such as reconstruction, reorganization, transformation, and adaptation, and evaluate the external and internal forces that cause the reduction of resilience at the same time. Therefore, on the one hand, resilience focuses on reducing the risk of people’s quality of life in critical and normal conditions, and on the other hand, it emphasizes on returning to the normal status through reducing vulnerability, adaptation, improving capacities and strengths, and sustainable development. In addition, different stages of resilience and its different approaches are mentioned with regard to (short, medium, and long term) time. Besides that, efforts were made to propose various tools, methods, and indices to measure the resilience. The focus of these researches has been on measuring the aspects of resilience, including economic, social, governance, health, technological, and environmental, and efforts have been made to measure the resilience in these aspects. Furthermore, following various challenges such as the growing trend of urbanization, extreme weather changes, economic crises, natural crises such as floods and earthquakes, humanitarian crises such as poverty, social segregation, and epidemics, researches are oriented towards the role of local communities in increasing resilience. In fact, the role of local communities in resilience, as a fundamental role to reduce vulnerabilities, provides a means with planners to measure resilience and identify communities that have low power in terms of response, recovery, capacities adaptation, and high vulnerability against crises^32^. Although measuring the resilience is important, its operationalization has always faced the challenge. In fact, indices utilization to measure their resilience and operationalization requires a good knowledge of the fundamental structures of local communities. Though various means have been proposed for the resilience of local communities, the combination of these results for the use of policy-makers and decision makers for the communities’ resilience still faces limitations^33^. In fact, it seems that the impact of the components influential on the resilience is different in societies and the importance of resilience aspects and its indices are also different. For example, Tariq et al.^34^ emphasize the five physical, health, economic, social, and governance aspects in the form of 86 indices for local resilience. Zhong et al.^35^ emphasize on prioritizing the promotion of science and technology over the economic-social situation and the environment built in local communities in Nanning, China. Pays attention to resilience from social-ecological-technological aspects. But the important note is that the components influential in resilience are different from one society to another according to the research conducted. It means that cities in coastal areas face different challenges^36^ in terms of resilience from the ones located in desert areas^37^. Furthermore, different economic systems of countries have different impacts on the resilience of local communities^38^. More than 50 tools have been proposed to measure resilience to natural and human hazards, which have posed challenges in explaining them in a new society^39,40^. Therefore, this study seeks to examine the concept of resilience in different institutional conditions. In fact, it seems that the institutional environment plays an effective role in realization of resilience in local communities. As an infrastructure for realization of other aspects of resilience, the institutional environment paves the way for its formation and realization in local communities. Then, the dynamics of the final environment can play an effective role in resilience creation.

Although the creation of resilient local communities has been proposed as an approach to overcome crises of human and natural origin, the creation of such communities has always been a challenge for Iran. In fact, the existence of political economy is known as one of the important factors in the formation and role-creation of local communities.

Political economy takes into account the relationship between politics and economics^41^ and the role of institutions and power relations, including the state as a basic political institution^42^ and is based on two fundamental principles. The first principle derives from the nature of social groups and second from the characteristics of state. According to the former, the interests of social groups are heterogonous that leads to the social contradiction derived from policy making and the latter refers to the state’s lack of impartiality towards social contradictions^43^. Urban political economy is very important and complex issue that is glaring in the cities and plays an active role in urban form, function, management, development and many urban challenges, especially in developing countries^44,45^. In spite of the importance of political economy analysis, most of urban studies are carried out without any coherent analysis of the power relations and the political economy nature^46,47^. It is necessary to take into account this fundamental issue to analyze any policy-making and sustainability discussions regarding process, etiology, hidden connections, structural drivers, determinants and constraints. One of the crucial areas that is directly and indirectly affected by political economy is local communities.

The political economy influence from macro to micro level in the decision-making system to implementation in the institutional environment has caused the absence or reduction of the role-creation of these societies. On the other hand, since Iran relies on oil income, it has a rent and quasi-capitalist economy, which has added to the challenges of creating resilient societies.

Thus, it can be said that any policy-making in the field of resilience is affected by political economy. The economical-political influence in the implementation and planning system has caused the role of local entities and non-governmental organizations to be greatly reduced or ignored in the implementation and planning system and the non-establishment of local entities in some cases. On the other hand, the government is actually not accountable to the local communities due to the government’s dependence on oil revenue and has proceeded to concentrate the power in the planning and political system instead of facilitating the grounds for the role-creation of local communities in increasing the resilience. In fact, the political economy in Iran has led to the formation of centralized power and a top-down planning system. Therefore, the question arises as to how the resilience of local communities can be realized with regard to the influence of political economy on the implementation and decision-making system. Therefore, the aim of the study is to explore how the political economy affects the local communities resilience and how it is related to institutional, economic, social, and ecological-physical environment variables. The secondary research objectives include:

- Identifying the obstacles to the formation and role-playing of local communities resilience, considering the impact of political economy
- Measuring the level of resilience in various dimensions, and
- Evaluating the physical vulnerability.

If we accept that the performance of resilience components differs based on the management-institutional structures in each society, then identifying these components and evaluating their impact according to the planning system at both macro and micro levels is essential. Identifying the urban planning system, examining the economic, social, and environmental factors at the city level, will clearly assist in understanding the function of effective factors in resilience. On the other hand, examining the residents’ perspectives as members of social networks will play an effective role in analyzing resilient communities. Consequently, in addition to systematic study of resilience, we need to examine effective factors and their function in sub-systems.

Alongside the impact of political economy on the resilience of local communities, Iran has faced various natural disasters in recent decades. Based on the studies conducted from 1990 to 2020, Iran has faced 247 major crises that have killed around 200,000 people and caused economic losses that have negatively affected the lives of 55 million people^37^. Limited researches have been conducted in the field of resilience despite the occurrence of these crises. The results of the few researches carried out are not effective due to not addressing the political economy. In fact, how to create resilient local communities considering the impact of political economy is a challenge that has not been addressed so far. The impacts of political economy have caused the formation of rent-based economy, which has had many consequences on other aspects of resilience. Neglecting the role of political economy has caused that the results of other researches on the resilience of local communities do not contribute significantly to the planning and policy-making system of Iran to create resilient local communities. Therefore, the research has investigated the impact of political economy on the resilience of the local community by selecting the central part of Zanjan as the case study sample and makes an attempt to address the obstacles to creating the resilience of local communities and the knowledge gap in this field by analyzing the political economy. Hence, the research questions are as follows:

- What is the mechanism of influence of political economy on the resilience of local communities?
- How much is the resilience of the central part of Zanjan considering its multiple aspects?

## Research methodology

### Identification of the study area

**Figure 1:**
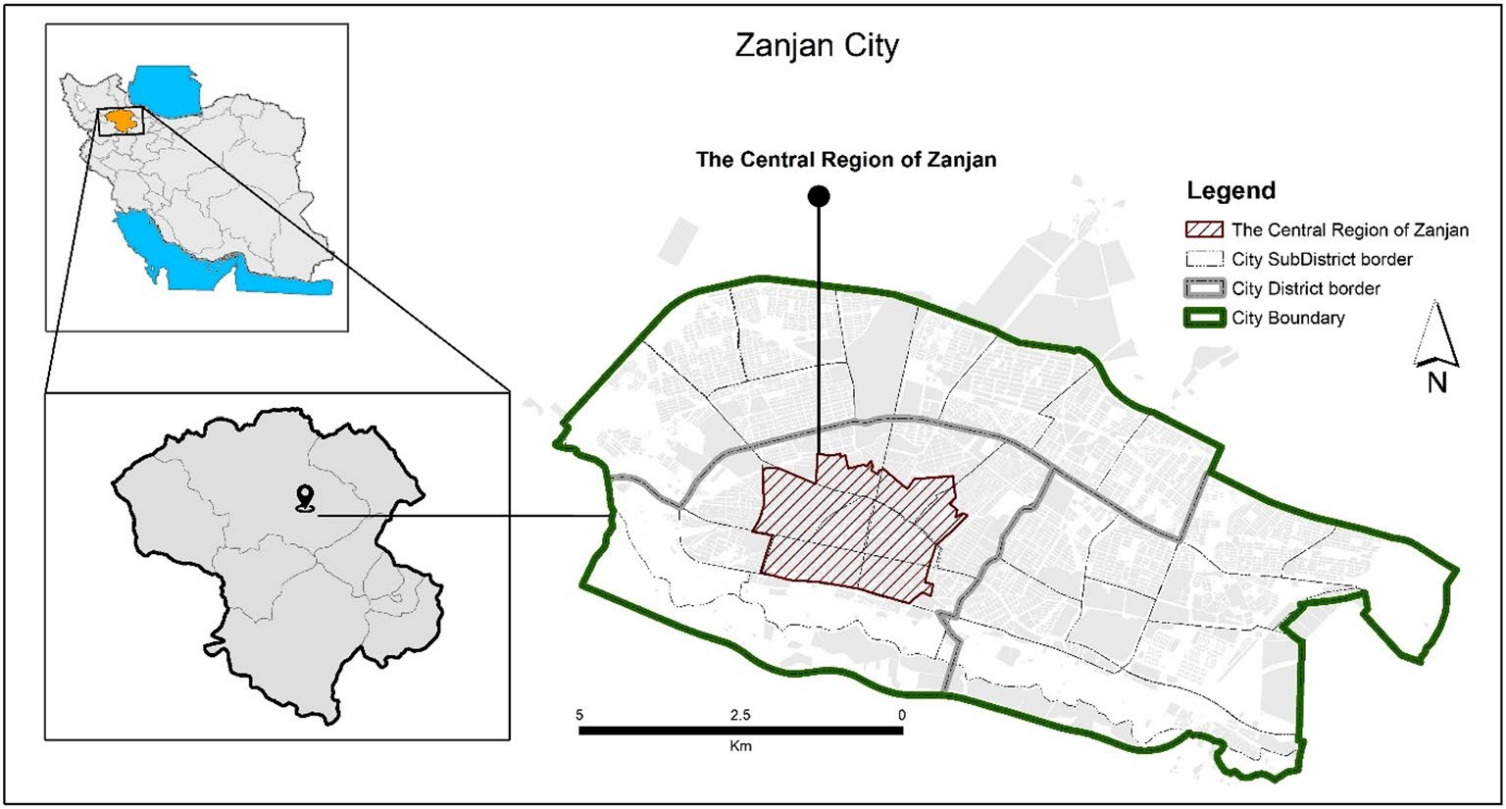
Authors, 2023. the geographical location of the central part in Zanjan city.

Zanjan is the capital of the province, with a population of 430,871 in 2022, with an average household size of 3.5. The city’s public participation rate is 40.8%, and the employment rate is 87.1%. The majority of the workforce, 76.69%, is engaged in the service sector, and 19.85% in the industrial sector, with an economic participation rate of 35.5%. The literacy rate is 87.55%, and the university education rate is 40.5%. Zanjan has a semi-arid and cold climate, with an altitude of 1659 m above sea level. Its annual average precipitation, temperature, and humidity are 295 mm, 10.9 degrees Celsius, and 54%, respectively. The average minimum temperature in the coldest month of “Bahman” is − 7.5 degrees Celsius, and the average maximum temperature in the hottest month of “Mordad” is 32.1 degrees Celsius. The temperature drops below zero for 118 days a year, with “Dey” and “Bahman” having the most days, with 27 days each. The wettest month is “Ordibehesht” with 52.5 mm of rainfall, and the driest month is “Shahrivar” with 3.5 mm of rainfall. The central part of Zanjan city, as shown in Fig. 1, is the primary nucleus of the city, covering an area of 347 hectares of the city’s total land area, with a population of 34,347 people and 9,062 households. More than 70% of the land use is residential, while the commercial land use is 32 hectares, due to the presence of the traditional market in the central part. The green space in this area covers 3.5 hectares, and the population density, according to the latest census in 2022 by the Statistical Center of Iran, is 106 people per hectare. Zanjan city is located in a point with the highest seismicity grade according to the Iranian earthquake regulation 2800. The old texture of Zanjan city, which is mostly located in the central part of the city, and the lack of necessary attention in previous constructions have made the city vulnerable in case of human or natural crises. This section has an economic position, including the existence of a large market and historical textures, which have identity and historical value for the whole city. On the other hand, the central part faces undesirable conditions in terms of physical and environmental aspects, including incompatible uses, lack of green spaces, improper distribution, old age, organic texture, use of low-durable materials in construction, and social, economic, and management problems. Therefore, in case of a crisis in this section, its impact will be twofold and can cause irreparable damage to the central part.

## Methodology

The research method is a mix method. For analyzing the institutional environment in relation to resilience, qualitative methods have been used, and for measuring vulnerability and resilience, quantitative methods have been employed, which are explained in detail below. In the first step, the management-institutional variables have been examined as a basis for facilitating the process of achieving social resilience. In addition to reviewing the documents and literature by experts to identify the obstacles to achieving social resilience, all the management organizations and institutions, especially at the local level, have been identified and their relationships have been explained. In the second step, before examining the resilience status of the study area, it is necessary to assess the physical vulnerability of the neighborhood in dealing with crises. The Delphi-Fuzzy method has been used to determine the vulnerability assessment criteria. Therefore, based on the experts’ opinions and global literature review^48–51^, criteria have been evaluated for assessing the physical vulnerability, and linguistic variables have been converted into triangular fuzzy numbers (), which are shown in Table 1.

**Table 1:** Triangular fuzzy numbers corresponding to linguistic variables.

Likert scale	Linguistic variables	Fuzzy numbers
5	Very high	0.1, 0.8, 0.6
4	High	0.8, 0.6, 0.4
3	Moderate	0.6, 0.4, 0.2
2	Low	0.4, 0.2, 0.0
1	Very low	0.2, 0.0, 0.0

After this stage, the validation and screening of factors or variables were performed. This was done by comparing the value of the acquisition value of each variable with the threshold value. In this study, based on the experts’ opinions and the number of variables, the value of 0.7 was considered as the threshold value. Triangular fuzzy values of the experts’ opinions were calculated and then, the fuzzy average of their opinions was calculated to calculate the average of n respondents’ opinions. The fuzzy number calculation for each of the factors was obtained using the following equations:

1 $${\pi }_{ij} = ({a}_{ij},{b}_{ij},{c}_{ij}) i = \mathrm{1,2}, ..., n j -= \mathrm{1,2}, ... , m$$

2 $${a}_{j}=\sum \frac{{a}_{ij}}{n}$$

3 $${b}_{j}=\sum \frac{{b}_{ij}}{n}$$

4 $${c}_{j}=\sum \frac{{c}_{ij}}{n}$$

where, the index *i* refers to the expert and index *j* refers to the decision factor. Also, the defuzzified values of the fuzzy number average were obtained using the following equation.

5 $$\mathrm{Crisp}=\frac{a + b + c}{3}$$

**Figure 2:**
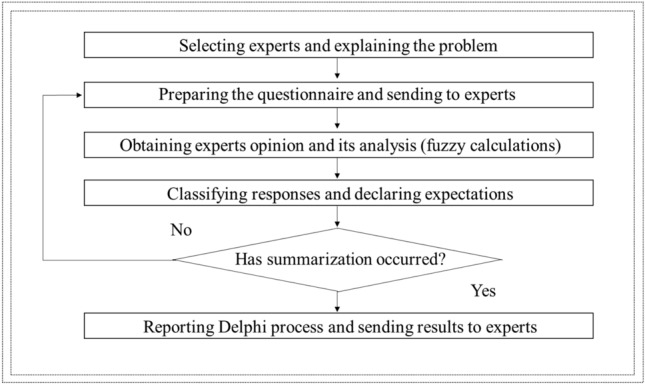
The Delphi-Fuzzy method implementation algorithm.

The Delphi-Fuzzy method implementation algorithm is presented in Fig. 2.

The hierarchical method and ArcMap software were used to measure the degree of physical vulnerability of the central part of the area based on spatial data. The five-step process included:

1. Classifying data based on resilience indicators.
2. Transferring data to ArcGIS and creating a geodatabase.
3. Creating a layer for each indicator and fuzzifying the layers based on the experts’ opinions.
4. Determining the impact coefficient of each indicator on resilience using the Analytic Hierarchy Process (AHP) model.
5. Overlaying the layers and applying the corresponding coefficients to prepare the final resilience map.

In the expert panel section, 25 experts were surveyed, and the data were entered into the Expert Choice software. According to the CR value of 0.009, which is less than 0.1, the experts’ opinions were deemed to be compatible. If CR ≤ 0.1 (agreement ratio), it indicates that the necessary compatibility has been observed in the judgments, and if 0.1 < CR, the judgments need to be reconsidered.

In the third step, based on the assessment of resilience status and the examination of residents’ views, a field survey was conducted. Therefore, the research method is survey and correlational. After identifying factors affecting resilience, to reduce the components for factor analysis, the KMO and Bartlett’s Test were used. The KMO value of 0.724 indicates that the data is reducible to a few underlying and fundamental factors. The Bartlett’s Test at the 0.1 level of significance shows that the correlation matrix between the items is not unitary. This means that there is a high correlation between the items within each factor, but there is no correlation between the items of one factor with the items of another factor (Table 2).

**Table 2:** KMO and Bartlett’s Test.

Kaiser–Meyer–Olkin Measure of Sampling Adequacy		0.724
Bartlett’s Test of Sphericity	Approx. Chi-Square	2341.677
	df	495
	Sig	0.000

After performing exploratory factor analysis, four components were selected as the main factors based on Table 3, which had the highest correlation with their subset items. It was determined that 25 items related to resilience could be reduced to four factors in this study. According to the eigenvalue table, the four factors above one explains about 71% of the variance of the resilience variable.

**Table 3:** Total variance explained.

Component	Initial Eigenvalues			Extraction sums of squared loadings		
	Total	% of variance	Cumulative %	Total	% of variance	Cumulative %
1	10.527	20.245	20.245	10.527	20.245	20.245
2	8.743	16.813	37.058	8.743	16.813	37.058
3	6.852	13.178	50.236	6.852	13.178	50.236
4	6.265	12.048	62.284	6.265	12.048	62.284
5	0.385	1.586	66.870	0.385	2.586	66.870
6	0.261	1.348	71.218	0.261	2.348	71.218
.	.	.	.			
.	.	.	.			
25	0.361	2.618	84.107			

Finally, the sample size was determined based on the Daniel formula (1995) after determining the indicators and questionnaire items for the central part of Zanjan population.

$$n = \frac{{Z}^{2}p(1-P)}{{d}^{2}}$$

where Z = the value of the standard normal variable (for a 95% confidence level, it is equal to 1.96). P = the expected ratio (in this study, it is considered as 20%, which is equal to 0.3). *d* = the precision (in this research, it is equal to 0.0392)

This formula has a correction that, if n/N is greater than 0.05, finite population correction (FPC) should be used. The corrected formula is as follows:

Where:

$$n = \frac{{NZ}^{2}p(1-P)}{{d}^{2}(N -1) + {z}^{2}P(1-P)}$$

*n* = sample size (with finite population correction), *N* = population size, *Z* = the value of the standard normal variable (for a 95% confidence level, it is equal to 1.96), *P* = the expected ratio, *d* = the precision

Based on the above assumptions and a 95% confidence level, the sample size according to the Daniel formula is 401, and since *N*/*n* ≤ 0.05, there is no need for the correction formula, and 401 is considered as the final sample size. To conduct the survey, 401 plates were selected from the statistical blocks’ map for personal visits. The sampling was done through simple random sampling and personal visits to households at three different times of the day to cover all age and gender groups. The research period was 14 days. One-Sample Test was used to measure the resilience status of the central area from the perspective of the residents.

**Figure 3:**
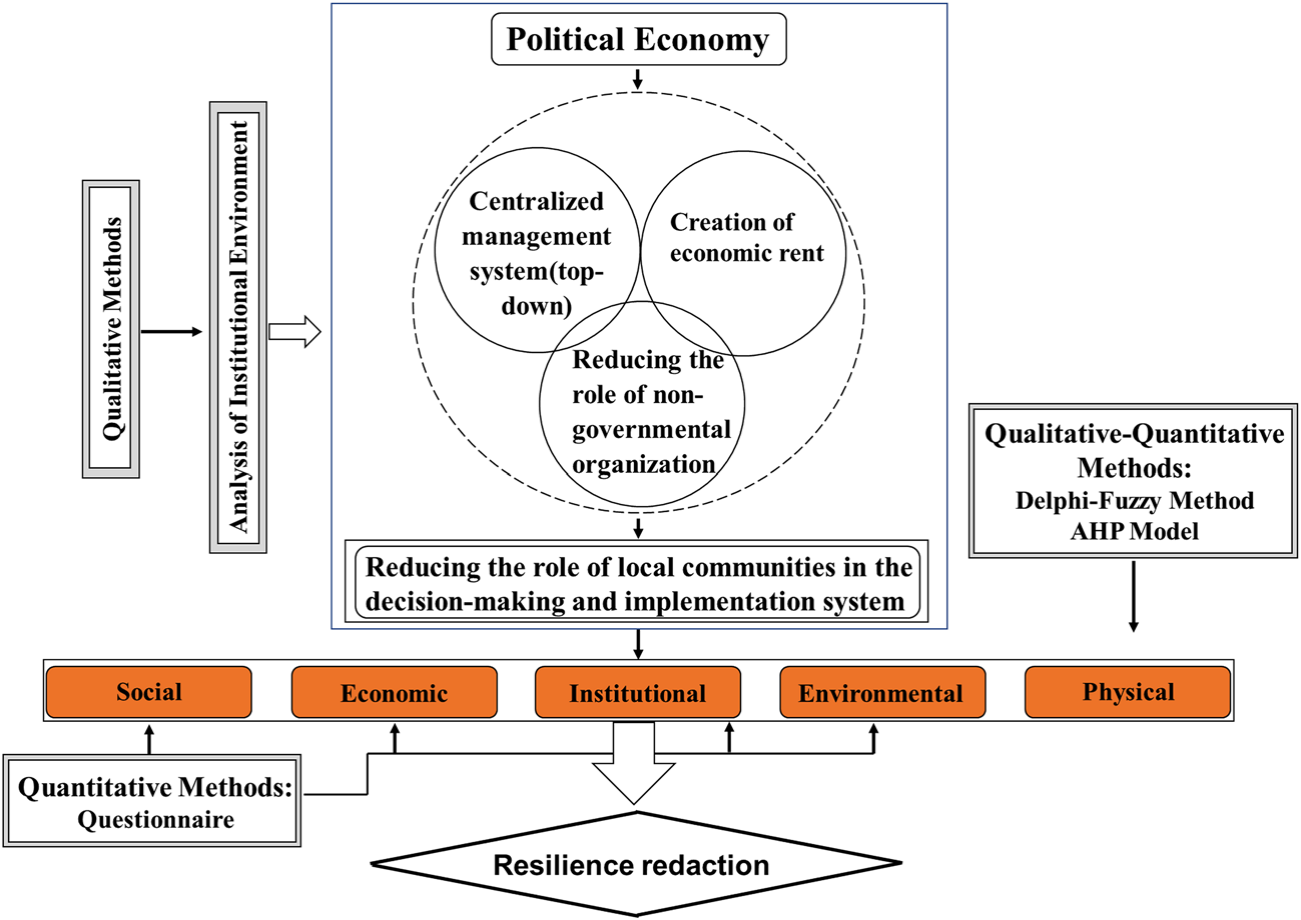
Technique routine chart to present the research path.

Figure 3 has been developed for a better understanding of the research route. In this figure, an effort has been made to specify research methodology used in each section in addition to the conceptual relationships among the research variables. A qualitative method was used to analyze the institutional environment and a quantitative–qualitative method was used using the Delphi-fuzzy model and AHP to measure the extent of physical resilience of the central part. In addition, the information has been gathered in the form of a questionnaire to measure other aspects of resilience from the perspective of the residents. The research is based on institutional environment analysis to indicate the influence of political economy in planning and policy-making. In fact, it has addressed how the political economy affects the resilience of local communities and emphasizes how the political economy causes the reduction of the role-playing of local communities in resilience.

## Results

### Resilience and institutional environment

**Figure 4:**
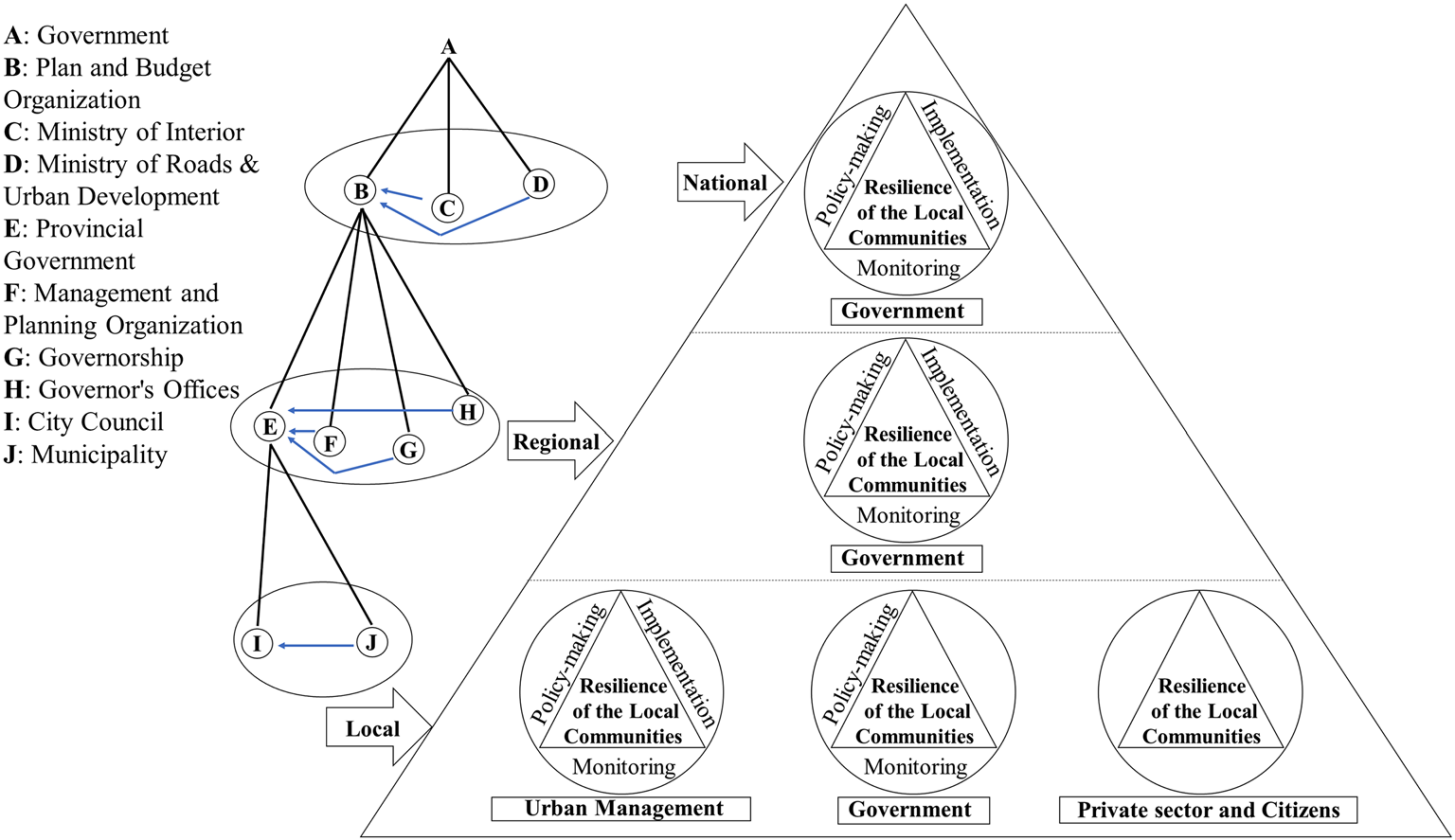
Institutions related to the planning system.

The management structure at the national level in Iran is centralized. In fact, all macro-level policies are formulated at the macro level and sent to lower-level sectors in the form of regulations and guidelines with mandatory implementation. According to Fig. 4, the Iranian government enforces policies through several ministries and organizations. In the regional sector, government institutions are responsible for policy-making and enforcement. The lack of local level in the planning system and the top-down approach are prominent features of the country’s planning system. At the city level, the management structure is in the form of a city council and mayor. The task of preparing and approving urban development plans is assigned to the Ministry of Roads and Urban Development, which is responsible for executive and supervisory tasks after approval by the municipality. Urban development plans are presented in the form of a comprehensive plan that outlines the general principles of urban development, and a detailed plan as an executive plan. The lack of local planning has resulted in the municipality being the only direct institution involved in planning, implementation, and supervision at the local level. The role played by this institution and the lack of mechanisms for private sector and resident participation have resulted in these two groups being excluded from all planning processes in practice. On the other hand, the government, as a provider of a portion of the resources for municipal income, refrains from financing that encourages illegal increases in building density for municipal income. This is while many policy-making researchers at the local community level emphasize the need to solve environmental, social, and economic problems and achieve community resilience^52^.

**Figure 5:**
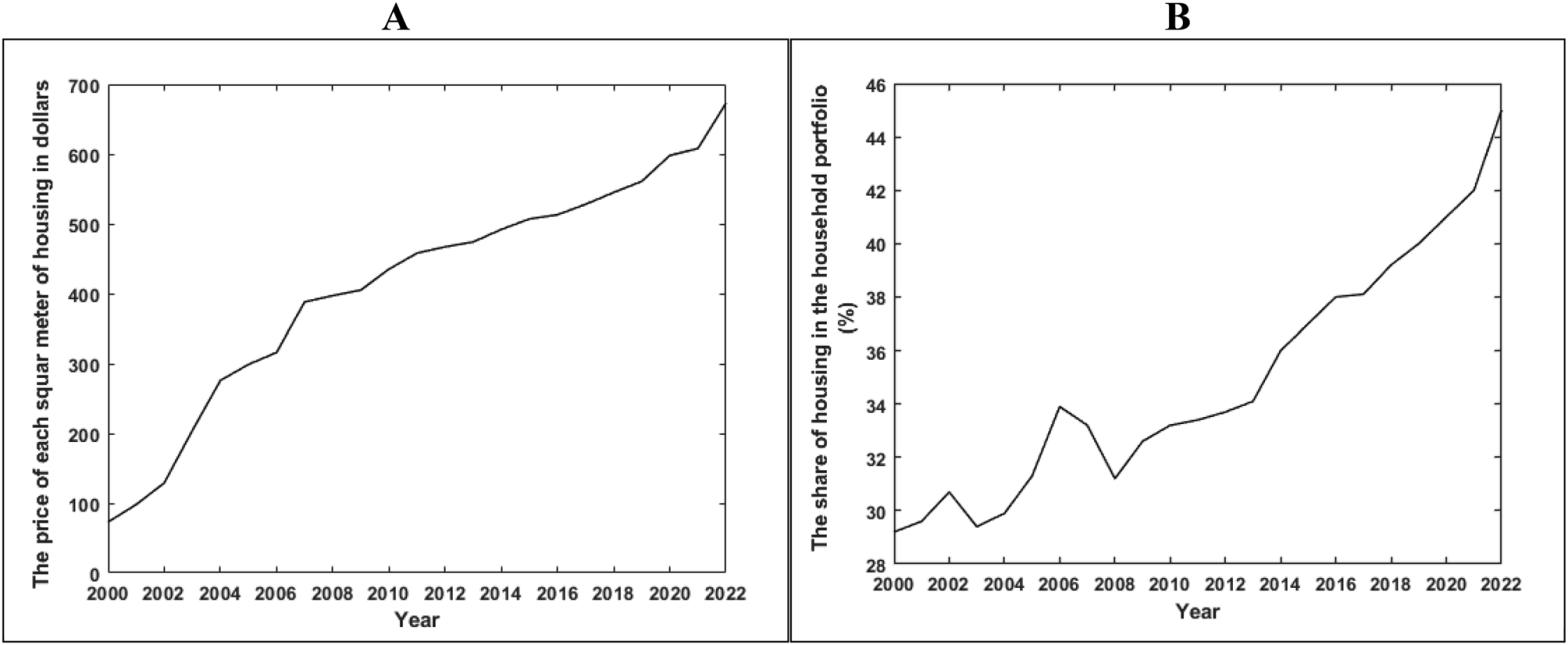
The price per square meter of housing in the central part of Zanjan in dollars (USD) (**A**). Share of housing cost in household portfolio in the central part of Zanjan (**B**).

**Figure 6:**
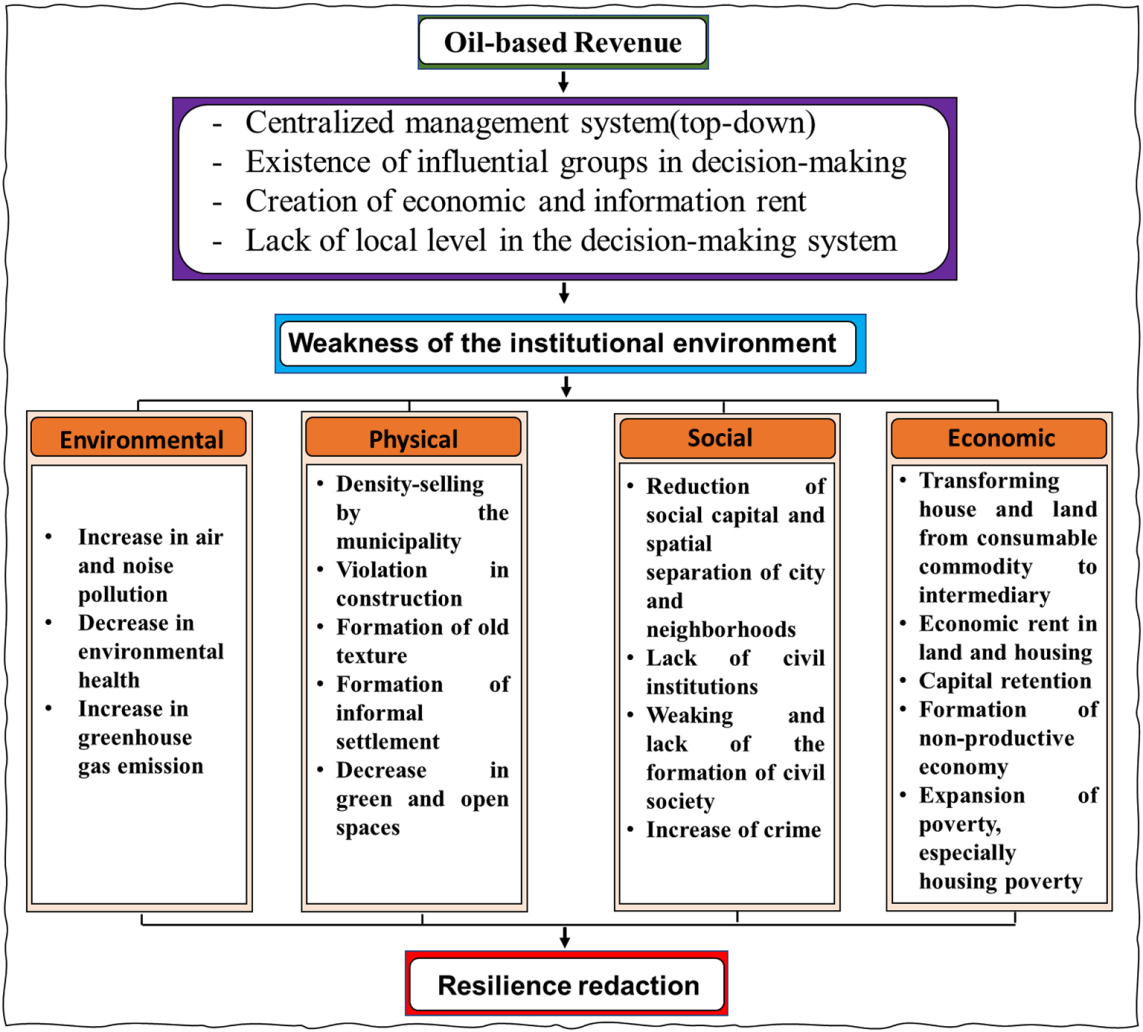
Factors affecting resilience reduction.

Given the managerial, economic, and political structure of the urban planning system, there is a need to design a framework to identify the factors and how each factor plays a role, taking into account the contextual variables shown in Fig. 6. The management-institutional structure is recognized as the main pillars in this area^53,54^. The centralized government structure does not allow for maximum participation of residents and the private sector. Therefore, decision-making and policy-making will not be oriented towards the public interest, and the local level has not been able to provide the residents’ interests, given its lack of connection with the national level^55^. Therefore, we need horizontal and vertical links between planning levels and the participation of government, private sector, and residents. On the other hand, there is a meaningful relationship between the political and social structure. Over time, the centralized planning system leads to the passivity of the public and private sectors. The result of this process is a decrease in capital and social cohesion among residents, which is considered one of the most important capacities of society for resilience^12^. In fact, increasing areas of collaboration among residents empowers them in the decision-making process and identifies the resilience status and increases it through social network formation^56^. In addition, the management-institutional structure is effective through construction laws and regulations on the ecological-physical basis. The existence of influential groups in decision-making and the lack of transparency in construction violations and density-selling for income have affected the environmental conditions of the neighborhood, resulting in an increase in building and population density, and ultimately, greater vulnerability of the neighborhood. On the other hand, with housing turning from a consumable commodity to a capital asset, household costs have increased, and a significant portion of income has been allocated to the housing sector. This has led to poverty, especially housing poverty, and the process of renovating and rebuilding the deteriorating fabric of the neighborhood faces serious challenges. The data of the Statistical Centre of Iran in Fig. 5 shows that the housing cost in the household portfolio in the central part of Zanjan has increased so that the share of housing cost in the household portfolio has reached from 29% in 2000 to 45% in 2022. This confirms that the share of affordable housing has experienced a decreasing trend and providing the housing for low-income groups has become out of reach. On the other hand, the amount of each square meter of housing has also increased as time passed. The housing price was about USD 73 per square meter in 2000 and reached USD 673 in 2022. This confirms the unproductive economy, which causes the capital to be locked up and paves the way for housing speculation. This can be mentioned as the impact of political economy on the housing economy that causes the reduction of the local community resilience in the long-term.

The following framework refers to contextual factors that can play a negative role in the resilience process. The negative effects of rent-seeking economics have led to the emergence of the Dutch disease in the economy. Due to the government’s reliance on oil revenues, which account for over 30% of the country’s budget, we are witnessing two types of reactions in the economic sphere regarding the decrease or increase in oil revenues. In the event of an increase in oil revenues, the government’s general budget also increases. However, in the economy with three components, due to the decrease in the exchange rate, imports become more cost-effective, and on the other hand, injecting income directly into the community increases liquidity. This leads to an increase in household income and an increase in demand in the economy. All these factors, considering the investment risk in other sectors and the weakening of domestic production, leads to the capital and labor force moving from the exchangeable goods production sector to the non-exchangeable goods production sector. This leads to an increase in investment in the housing sector because housing is not only considered a consumable commodity but also plays a profitable role. These factors have led to the emergence of the Dutch disease in the housing market in Iran in some years. On the other hand, with the decrease in oil revenues, the government’s general budget decreases. Therefore, the government is inclined to increase revenues through direct and indirect taxes. In addition, due to the decrease in income, we are witnessing a decrease in the growth rate of liquidity in society. Therefore, the overall consumer demand in the economy decreases, which leads to a decrease in investment in the housing sector and a tendency to invest in other sectors of the economy if suitable conditions are provided. This somewhat reduces profit-seeking demands in the housing sector and stabilizes housing prices (Fig. 6).

### Resilience measurement in the central district of Zanjan

To determine the criteria for measuring the level of resilience based on the Delphi-Fuzzy model, Table 4 has been extracted. This table provides a framework for evaluating the resilience status of the central district of Zanjan based on the Delphi-Fuzzy model, using nine criteria. The final score of the resilience level for various sectors has been evaluated based on the spectrum presented in Table 1.

**Table 4:** Evaluation criteria for the resilience of the central district of Zanjan.

Criteria	Sub-criteria	Very high	High	Moderate	Low	Very Low
Type of structure (C1)	Mud brick and wood					0.012
	Brick and wood				0.128	
	Brick and iron			0.518		
	Concrete		0.602			
	Metal frame	0.865				
Building age (C2)	More than 30 years old					0.078
	20 to 30 years old				0.144	
	10 to 20 years old			0.496		
	5 to 10 years old		0.644			
	Less than 5 years old	0.907				
Building quality (C3)	Dilapidated					0.069
	Demolition				0.150	
	Maintainable			0.501		
	Restored		0.629			
	New	0.872				
Number of floors (C4)	More than 4 floors					0.092
	4 floors				0.147	
	3 floors			0.509		
	2 floors		0.741			
	1 floor	0.881				
Occupancy level (C5)	80% to 100%					0.073
	60% to 80%				0.132	
	40% to 60%			0.498		
	20% to 40%		0.789			
	0% to 20%	0.845				
Parcel size (C6)	Less than 100 square meters					0.056
	100 to 200 square meters				0.116	
	200 to 300 square meters			0.511		
	300 to 400 square meters		0.701			
	More than 400 square meters	0.909				
Facade materials (C7)	Without facade					0.074
	Black and composite cement				0.125	
	White cement			0.529		
	Stone		0.734			
	Brick facade	0.820				
Building density(C8)	More than 240%					0.098
	120% to 240%				0.183	
	80% to 120%			0.532		
	40% to 80%		0.741			
	Less than 40%	0.921				
Population density (C9)	More than 400 people per hectare					0.063
	300 to 400 people per hectare				0.155	
	200 to 300 people per hectare			0.548		
	100 to 200 people per hectare		0.708			
	Less than 100 people per hectare	0.956				

**Figure 7:**
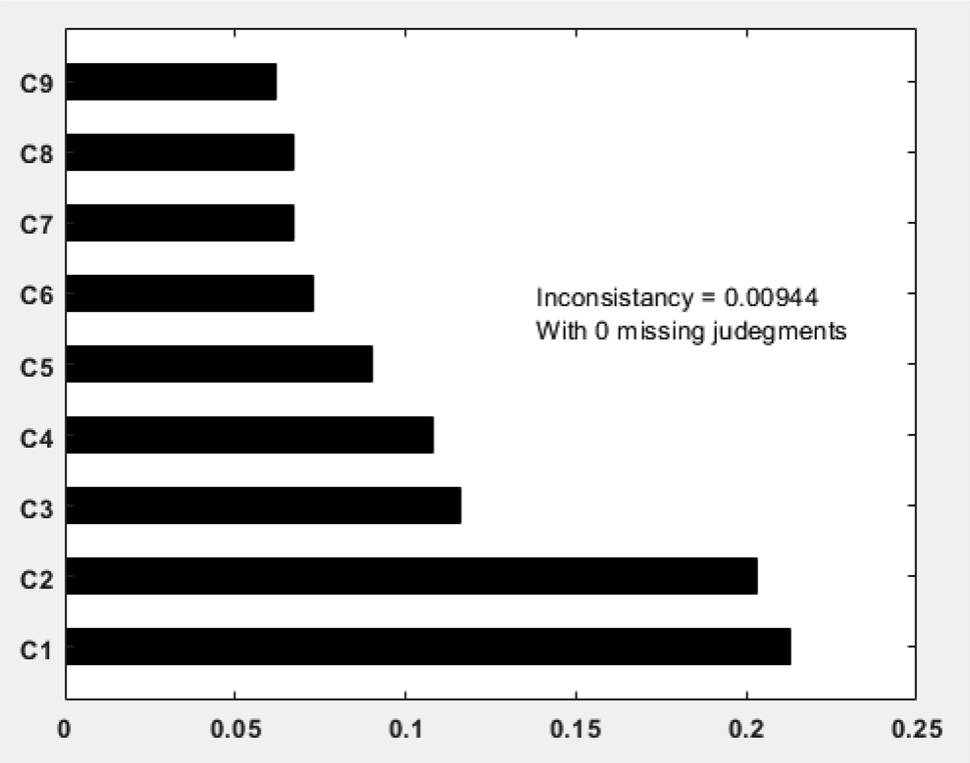
Importance of indicators for resilience based on the AHP model.

At this stage, the weighting of the main criteria was done using binary comparison method based on the experts’ opinions. The importance of each criterion was evaluated relative to each other. According to the output results from Fig. 7, the type of structure and building age were identified as the most influential factors in reducing damages caused by crises.

**Figure 8:**
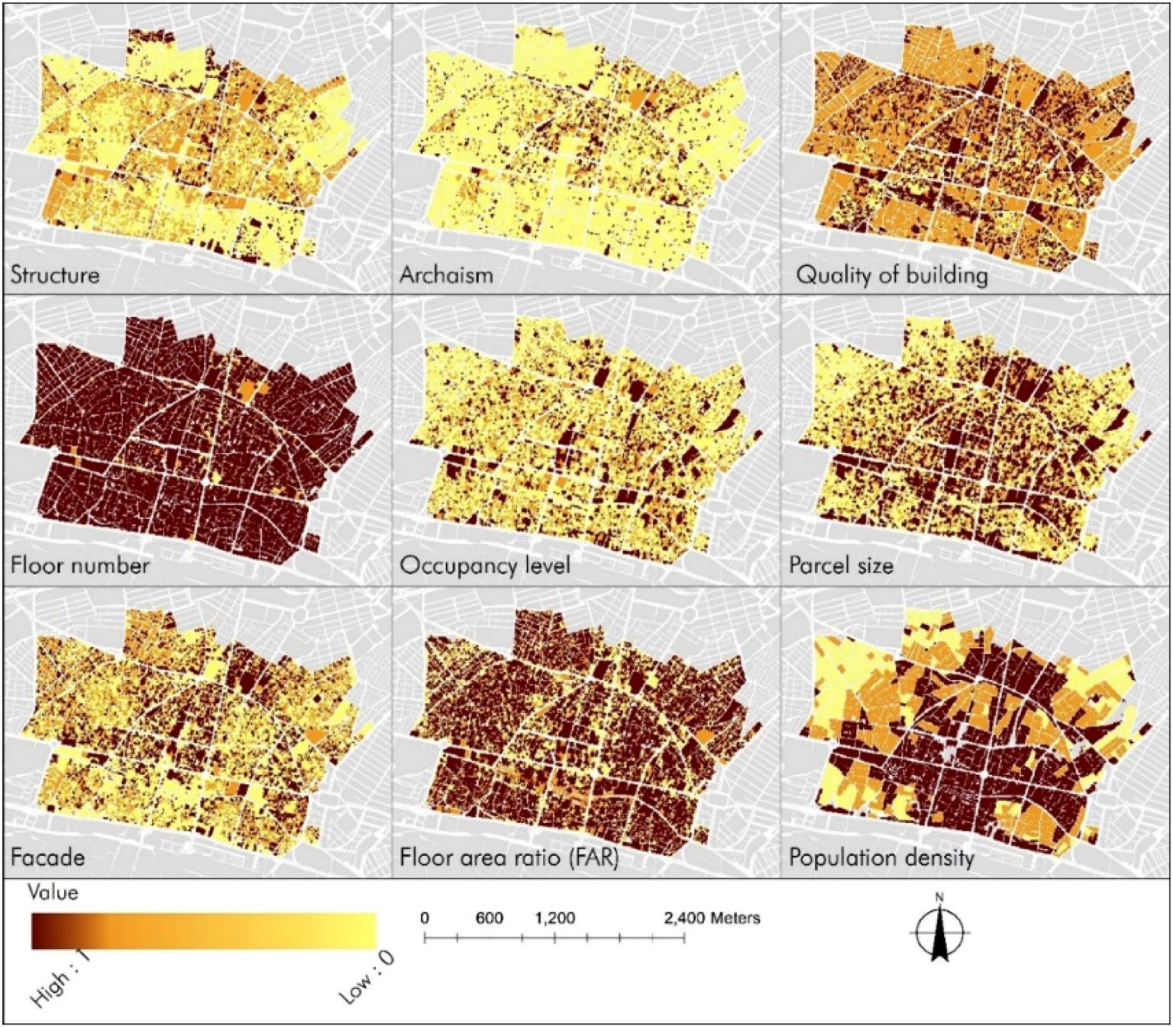
Resilience indicators status in the central district of Zanjan.

After weighting in the binary table and obtaining the weights of each of the 9 parameters of the Raster layers, we proceed to the process of combining the layers. Based on the final weights obtained from the results of the AHP table with an acceptable consistency coefficient of less than 0.1, the Classify tool is used for classification and the Raster Calculator is used to assign scores to each layer. The output of each layer is presented as a map in Fig. 8.

Based on the maps presented in Fig. 8, among the 9 criteria for determining vulnerability, three criteria including the number of floors and building and population density are at the highest risk of being affected by crises, which can increase vulnerability in case of a crisis. On the other hand, the criteria of the type of structure and building age show less vulnerability.

**Figure 9:**
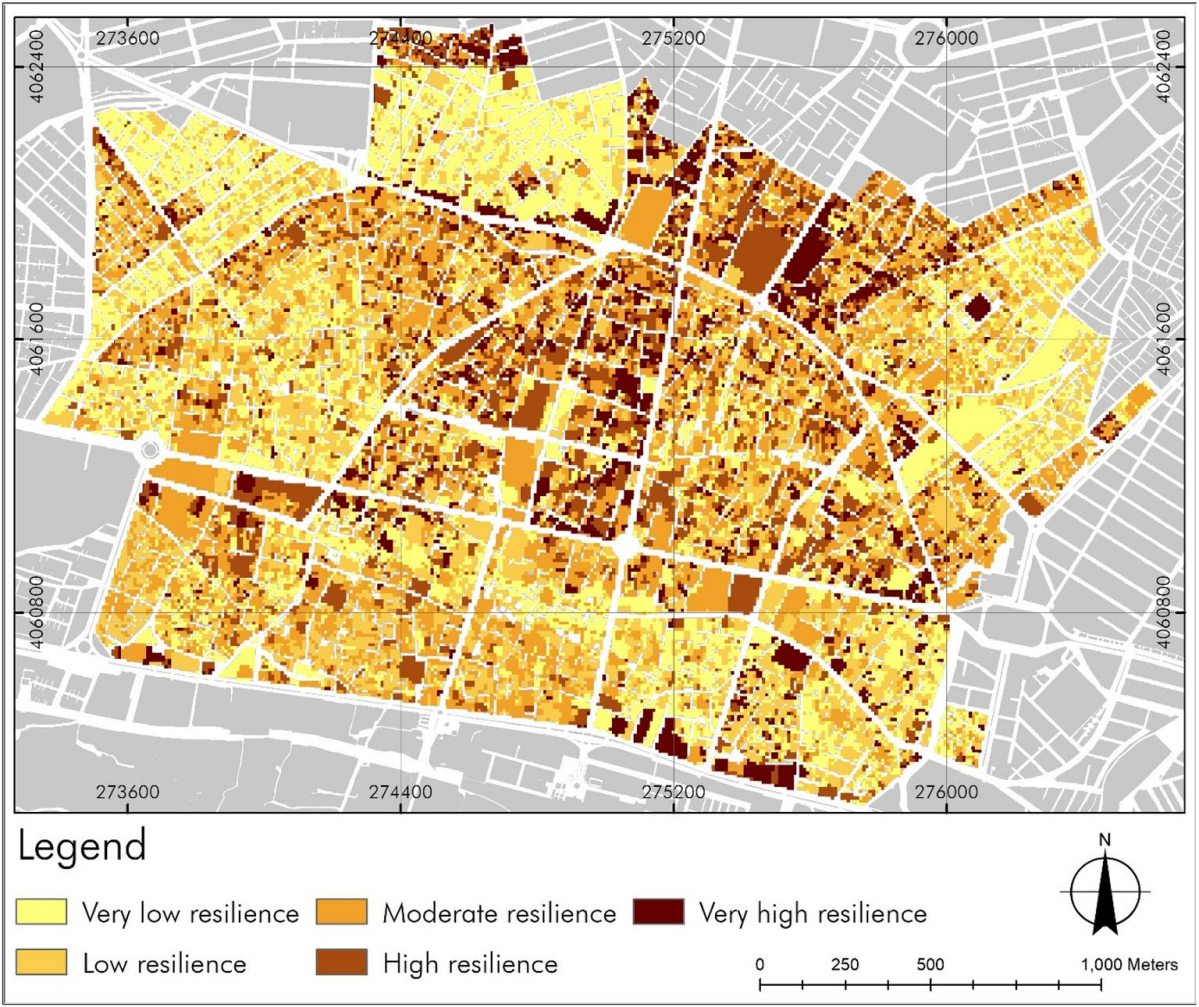
Resilience level of the central district of Zanjan based on 9 criteria.

The output of the model based on Fig. 9 and Table 5 shows that about 25% of the area in this district is highly vulnerable. Additionally, about 27% of the central district will face moderate vulnerability during crises. The vulnerability map indicates that out of the total area of 347.47 hectares, 87 hectares, which mostly have high building density and an aging texture, are in a high vulnerability category. Along with the issue of aging textures, which is due to the financial incapability of the residents for renovation or reconstruction, the problems and issues such as building code violations have also caused serious damage. The transformation of housing into a commodity has practically made affordable housing unattainable for most households in the area, and collaborative reconstruction plans have also become beyond the financial capacity of households. This has led to further deterioration and vulnerability due to the lack of desirable policies for reconstruction, rehabilitation, and renovation.

**Table 5:** Statistics of deteriorated textures resilience.

Percent	Area (ha)	Resilience
22.18	77.07	Very low
25.76	89.51	Low
27.05	94.00	Moderate
16.26	56.50	High
8.75	30.39	Very high

### Resilience measurement dimensions

The results of the descriptive analysis indicate that in the central district of Zanjan, the majority of respondents are women, forming 57% of the sample population. More than 50% of the respondents are under the age of 45, of which 36% are single and 63% are married. With regards to employment status, 19% of the respondents are unemployed, 11% are seasonal workers who are employed during certain times of the year, 32% are homeowners, and less than 40% of the respondents have a permanent job and monthly income. In this regard, the central district of Zanjan faces a lot of economic problems. More than 40% of the residents have lived in the area for over 30 years. Table 6 shows that there is a higher tendency for participation and connection with neighbors among the respondents in the social dimension. This means that there is potential social capacity, but this capacity has not been realized due to structural barriers in the social sphere, such as the lack of civil institutions and the absence of a local community with the power to make local decisions. On the other hand, knowledge and awareness of various crises are also at a lower level. In the economic dimension, the conditions are not desirable. The economic conditions of households have been affected by the economic crisis resulting from economic sanctions, the spread of the COVID-19 disease, and severe weaknesses in macroeconomic indicators (inflation, money supply growth, and increased unemployment), which have led to a reduction in economic resilience.

**Table 6:** One-sample test.

Dimensions	Indicators	Test Value = 3				
		t	Df	Mean	Sig. (2-tailed)	Mean Difference
Social	Adaptability to stress and turmoil	24.509	400	2.93	0.000	0.941
	level of participation in crisis resolution	15.096	400	3.35	0.000	0.588
	local perception of risk	20.072	400	3	0.000	1.000
	social tendency to participate	1.184	400	3.68	0.038	0.059
	participation in decision-making	11.700	400	2.89	0.000	0.559
	social connections with neighbors	23.850	400	3.89	0.000	0.706
	knowledge and awareness of crises	69.277	400	2.88	0.000	1.059
Economic	Capacity or ability to compensate for damages	18.400	400	2.77	0.000	0.588
	chances of obtaining a job	23.991	400	1.65	0.000	1.000
	families’ ability to return to employment conditions	53.403	400	2.5	0.000	1.118
	financial support from government and local institutions	12.113	400	2.59	0.000	0.382
	financial capacity of residents to participate	21.150	400	2.51	0.000	0.971
	use of financial and banking credits	19.566	400	2.22	0.000	0.618
Institutional	Education and implementation of maneuvers	25.658	400	1.85	0.000	0.735
	residents’ relationships with local institutions	22.431	400	3.37	0.000	1.029
	responsibility of institutions	28.387	400	2.56	0.000	1.000
	residents’ satisfaction with institutions’ performance	5.621	400	2.78	0.000	0.118
	activities of volunteer groups	25.658	400	3.37	0.000	0.882
	financial or technical incentives	18.400	400	2.54	0.000	0.588
	ease of street network and transportation	12.113	400	1.8	0.000	0.382
Environmental	Damages caused by natural hazards	23.991	400	3	0.000	1.000
	attention to climate in construction	26.633	400	2.76	0.000	0.853
	cleanliness and environmental sanitation	24.980	400	3.89	0.000	0.912
	management and disposal of surface waters	33.052	400	2.2	0.000	1.088
	usability of green space	− 7.839	400	3.45	0.000	− 0.206

The institution that appears to be considered as the foundation for other dimensions is also not in favorable conditions. Top-down planning and lack of planning at the local level, especially at the neighborhood level, have led only government and government-affiliated institutions to plan in this area. In fact, other public and private institutions play a very minor role in the development of the study area. The municipality, as a semi-public institution, is the only active organization in this sector. Such a process is evident in education and the level of responsibility of relevant institutions, which have the lowest level compared to other indicators. In the environmental dimension, due to high building density and aged texture, public transportation facilities have not expanded in this area, and the street network does not meet the necessary standards. Also, in terms of infrastructure, due to inappropriate management of surface waters, environmental health is also undesirable. Finally, a non-serious and indifferent attitude towards the crisis by the residents of the central district, weaknesses in informing relevant organizations about the necessary education on crisis preparedness, lack of attention to specific groups in the issue of facing crises, economic problems, and distrust of institutions related to renovation and reconstruction, and similar issues have led to a decrease in social resilience.

## Discussion

**Figure 10:**
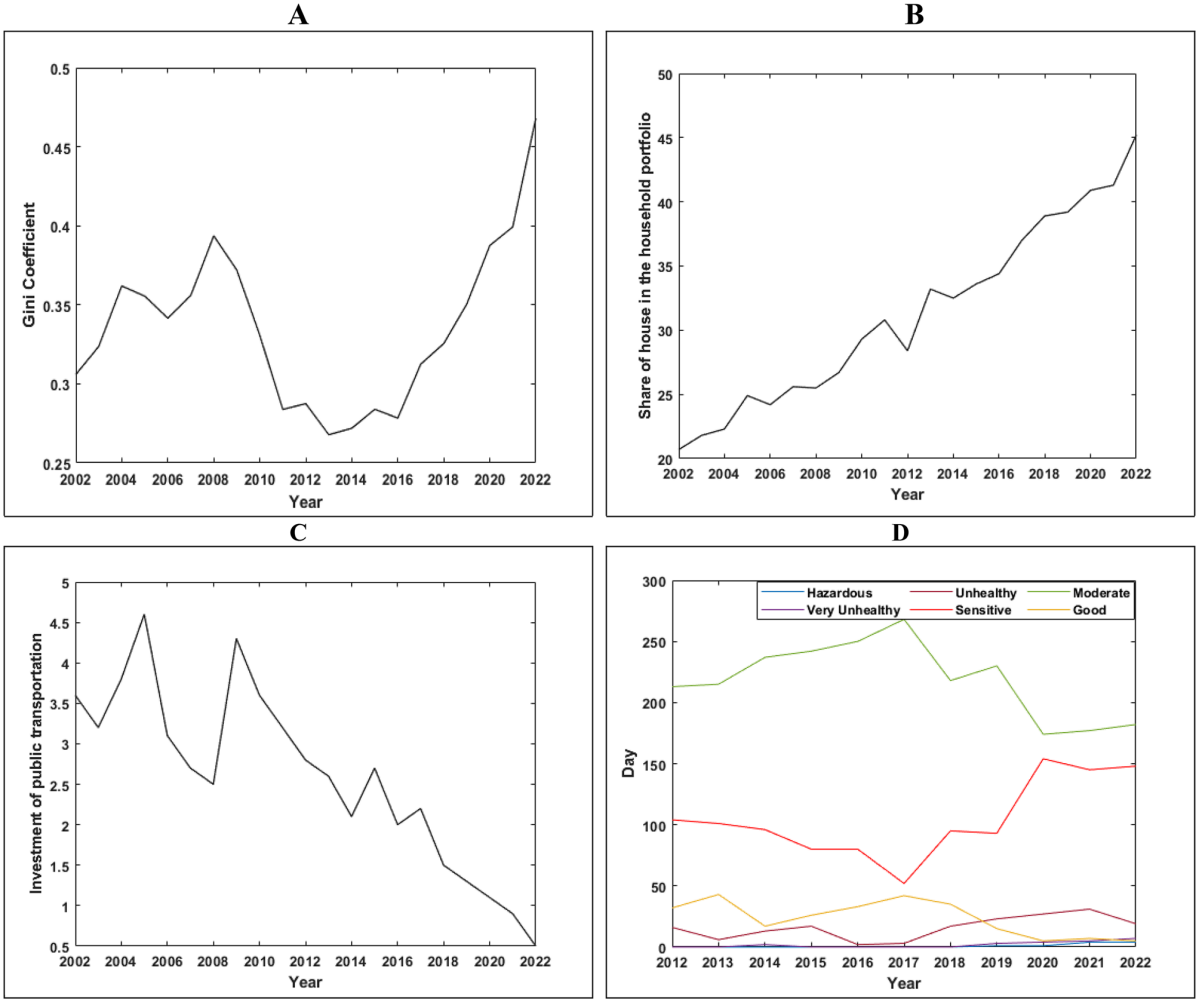
The Gini coefficient in Zanjan city (**A**), The share of housing costs in the household basket (**B**), The amount of investment in infrastructure and public transportation facilities by the municipality (**C**), The air quality index (**D**).

World literature studies indicate two general categories regarding the discussion of resilience. The first category is the evaluation of resilience from an ecological perspective, which emphasizes the analysis of systems and ecosystems^1,17–19^. The second category is related to studies in the field of social resilience. Resilience from individuals to society, especially in the psychological dimension, focuses more on social environment issues and the threshold of individuals’ tolerance. Another aspect of the discussion is the ecological-social approach, which, in addition to emphasizing environmental issues, also emphasizes the social environment and considers resilience in the cohesion of the human society with ecosystem services and resources for social sustainability. It aims to use the models and methods used for ecological resilience in social issues as well^20–26,57^. However, this approach also faces a serious challenge. The first challenge is that ecological resilience does not necessarily lead to social resilience. On the other hand, some researchers believe that the mechanisms of environmental system elements cannot be generalized to the social system. Although there are similarities between them, the feedback process of the social system is influenced not only by the structure but also by the actors^27^. In addition to resilience in ecological terms, all research has focused on the ability of the system to recover after disruption, but in social resilience, crises are identified as opportunities for change and development^28^. Some researchers consider the requirements of resilience to be linked to social, economic, and environmental capital and believe that addressing these three paths of capital will increase resilience in society^10,29^. Most studies have defined resilience indicators in land planning and use^58,59^, resilient construction^60,61^, functionality and permeability of functions ^62–64^, resources ^65–67^, financial incentives and financing^25,68–70^, and social capacity^11,71–73^. Although these indicators reflect the level of resilience in communities accurately, in countries like Iran, whose political economy and institutional environment are derived from it, it still plays a significant role in creating resilience or vulnerability. In fact, the political economy plays a fundamental role in policy-making and planning in the governance and management of cities. The performance of policies influenced by the political economy has led to the formation of land and housing rent-seeking and the imprisonment of capital in this sector. This has caused an increase in housing costs in the household basket and has led to an increase in urban poverty, housing poverty, and spatial segregation. The examination of urban poverty and housing costs in the household basket based on Iranian Statistical Center data in Fig. 10A,B shows an upward trend during the years under review, which is one of the consequences of the impact of the political economy on urban policies, which manifests itself in cities with an increase in urban and housing poverty. On the other hand, the impact of the political economy on public transportation development is also evident. The amount of investment by the Zanjan Municipality in public transportation during the years under review has been declining, and instead, personal car transportation has developed, as shown in Fig. 10C. In other words, due to the dominance of the political economy in the planning space, cities have developed towards car-centered development. The effects of these policies have a direct impact on climate change and air pollution. The examination of the air pollution control station in the central part of Zanjan in Fig. 10D shows that the trend of clean days has been declining during the years under review. On the other hand, the number of dangerous and very unhealthy days for all residents has been increasing. Therefore, it can be stated that all the aforementioned factors have directly caused a decrease in social resilience in the central part of Zanjan. The impact of the political economy has even been effective in macro policies. So that the effect of these policies in the long run has led to the formation of economic and political sanctions, which with the spread of the coronavirus put extra pressure on the social structure. In fact, the dual impact of sanctions and the coronavirus has left severe blows on reducing the resilience of cities, especially in the central part of Zanjan.

## Conclusion

Assessing resilience is a new and evolving research field and practice. Following the emergence and increase in various natural crises and their destructive impacts, especially in cities, new concepts were formed in response to these crises. In fact, the formation of the concept of local communities’ resilience was a response to the emerged crises. Over the past few decades, a growing number of studies have developed frameworks, methodologies, and conceptual indices for measuring resilience. Many of these conceptual frameworks, methodologies, and sets of indices have been proposed to measure the resilience characteristics of communities. However, the use of these indices requires an understanding and analysis of the institutional environment in each society. Although various researches have addressed how to create and measure a resilient community, the results of this research have faced limitations for planners and policy-makers. In fact, not paying attention to the role of political economy in Iran prevents the desired use of the practical results of other researches for the planning system. On the other hand, Iran is one of the most dangerous countries from the viewpoint of natural crises in which it is an undeniable necessity to deal with the issues such as resilience. Therefore, this study aimed to identify the resilience status of the central part of Zanjan based on the institutional environment and the impact of the political economy on this environment. In fact, this study seeks to explain the role of political economy and its impact mechanism on local communities for role-playing and influence on the resilience. The results of the study showed that the resilience status of this area of the city is relatively low. Physical degradation has also contributed to the further reduction of resilience. One of the main obstacles to increasing resilience is the impact of the political economy on the institutional environment. Its impact is evident in all decision-making and policy-making levels. For example, the formation of rent-seeking economics and its expansion into cities has led to the formation of land and housing rent-seeking, an increase in urban poverty, housing poverty, and spatial segregation. The impact of the political economy on the institutional environment has led to centralized and top-down planning and a lack of local community influence in the policymaking and decision-making system. These conditions have led to a decrease in the sense of belonging and self-alienation of residents and have challenged the solidarity of the community during crises. In addition, it has negative impacts on other aspects of local communities. Altogether, these factors cause the reduction or lack of the role of local communities in resilience. In conclusion, the resilience of societies in countries like Iran, with rent-seeking economies and political economy dominance in all levels of policymaking and planning systems, requires a transformational and evolutionary institutional group to utilize local opportunities and resources (human, physical, cultural, intelligence, and financial) and a decentralized and bottom-up planning system. According to the results of the research, it was determined that the political economy plays an important role in the failure of the formation of local communities’ resilience. Hence, the problem is formed in the mind that how the resilience of local communities is formed in such conditions or the societies like Iran, or what requirements, methods, or frameworks we need to create resilient communities. It seems that this problem can be a topic for another research.
